# Microbiota as a regulator of brain vulnerability across lifespan and disease contexts

**DOI:** 10.1186/s12974-026-03824-0

**Published:** 2026-04-22

**Authors:** Maryline Santerre, Natalia Shcherbik, Bassel E Sawaya

**Affiliations:** 1 Lewis Katz School of Medicine, FELS Cancer Institute for Personalized Medicine, Temple University, 3307 North Broad Street, Philadelphia, PA 19140 USA; 2 Department of Cell and Molecular Biology, School of Osteopathic Medicine, Rowan University, Stratford, NJ 08084 USA; 3 Department of Cancer and Cellular Biology, Lewis Katz School of Medicine, Temple University, Philadelphia, PA 19140 USA; 4 Department of Neural Sciences, Lewis Katz School of Medicine, Temple University, Philadelphia, PA 19140 USA

## Abstract

The gut microbiota influences brain health through inflammatory and metabolic pathways that regulate neurological vulnerability rather than directly causing specific diseases. This framework positions altered microbial composition as one among many environmental factors that modulate brain resilience across aging and disease contexts. Chronic low-grade inflammation driven by compositional and functional changes in the gut microbiota, intestinal barrier dysfunction, and alterations in bacterial metabolites contributes to systemic immune activation, affecting blood-brain barrier integrity, microglial function, and neuronal stress responses. These mechanisms operate across neurodegeneration, viral-associated cognitive decline, and brain tumor progression without constituting primary disease triggers. Aging amplifies microbiota-mediated inflammatory effects through progressive loss of microbial diversity and increased intestinal permeability. Evidence demonstrates associations between altered microbial composition and brain pathology, though these relationships reflect shared inflammatory pathways rather than direct microbial causation and are substantially confounded by diet, medication use, geographic variation, and disease-related behavioral changes. The vulnerability threshold framework proposed here is distinct from multi-hit models: rather than constituting an independent pathogenic insult, microbiota-derived signals modulate the quantitative threshold at which a given level of neurological stress produces clinical disease expression. This vulnerability-threshold model generates testable predictions: microbiota normalization should delay but not prevent neurodegeneration in high-genetic-risk individuals, and the severity of microbiota-associated inflammatory burden should predict the rate of progression rather than disease identity, predictions that longitudinal intervention trials can now begin to test.

## Overview

The rising global burden of neurological disease has exposed limitations in reductionist models of brain pathology that focus on isolated molecular pathways or singular cellular mechanisms. Despite decades of efforts targeting protein aggregation, oxidative stress, and neuroinflammatory cascades, therapeutic success in neurodegenerative diseases remains limited. These challenges suggest that brain disorders may reflect failures of systemic biological resilience rather than discrete pathogenic events. Increasing evidence supports the view that neurological health emerges from dynamic interactions among the nervous system, immune networks, metabolic regulation, and environmental biology. Within this broader systems framework, the microbiota–gut–brain axis has gained attention as a regulator of host physiology, not as a primary cause of neurological disease, but as a modulator of neurobiological vulnerability across the lifespan and in response to environmental exposures.

## Introduction

The brain does not fail in isolation. Neurodegenerative diseases, including Alzheimer’s disease (AD), Parkinson’s disease (PD), and amyotrophic lateral sclerosis (ALS), affect more than 50 million individuals worldwide and are characterized by progressive neuronal loss and accumulation of misfolded proteins [1]. Yet decades of therapeutic strategies centered on brain-intrinsic mechanisms, targeting protein aggregation, oxidative stress, and neuroinflammatory cascades, have yielded limited clinical benefit [2]. This persistent therapeutic failure suggests that neurological disorders may reflect failures of systemic biological resilience rather than discrete brain-intrinsic pathogenic events. Understanding why some individuals cross the threshold into neurodegeneration while others do not require a framework that extends beyond the brain itself.

Resilience and vulnerability in the nervous system are shaped by dynamic interactions among neural, immune, metabolic, and environmental systems across the lifespan. Within this broader systems framework, the gut microbiota has emerged as a powerful modulator of neurobiological vulnerability, not as a primary cause of neurological disease, but as a regulator of the systemic conditions under which the brain either maintains homeostasis or succumbs to pathological stress. The human gastrointestinal tract harbors approximately 100 trillion microorganisms, comprising over 1,000 bacterial species, collectively weighing 1–2 kg and encoding a gene repertoire that far exceeds that of the human genome [3]. This microbial ecosystem shapes immune tone, metabolic homeostasis, and neural function well beyond its digestive role [4], communicating with the central nervous system through vagal signaling, immune mediators, microbial metabolites, and modulation of blood-brain barrier integrity [5].

The significance of this gut-brain communication axis becomes apparent when neurological disease is examined through a vulnerability lens rather than a causation lens. Gastrointestinal symptoms precede overt neurological decline in several disorders [6, 7], suggesting that peripheral changes interact with central vulnerability long before clinical manifestation. Experimental models demonstrate that germ-free conditions attenuate α-synuclein pathology in PD paradigms and that microbiota manipulation meaningfully influences disease phenotypes [8]. Mechanistically, gut-altered microbial composition engages convergent pathways, including the production of immunogenic molecules such as lipopolysaccharide, modulation of systemic and central inflammatory signaling, alterations in metabolite profiles that affect neuronal energy metabolism, and disruption of blood-brain barrier integrity [9], collectively lowering the threshold for neurological disease expression.

**Fig. 1:**
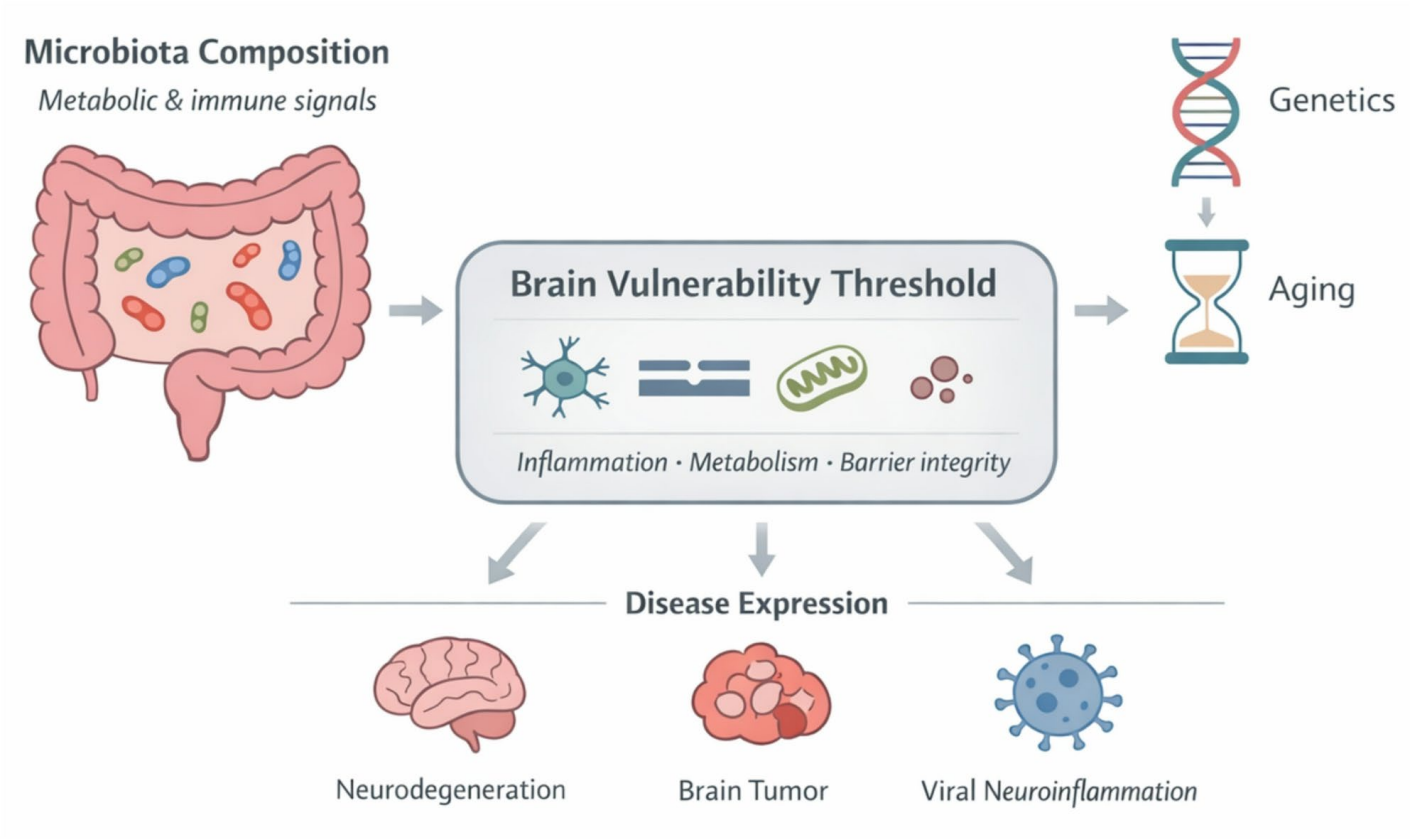
Microbiota regulation of brain vulnerability across lifespan and disease contexts. Alterations in gut microbiota composition influence systemic immune and metabolic tone. These signals converge on key regulators of brain resilience, including microglial activation state, barrier integrity, and mitochondrial function, thereby modulating a central vulnerability threshold. In the context of genetics and aging, this threshold determines susceptibility to diverse neurological conditions, including neurodegeneration, brain tumors, and viral CNS injury

This review proposes a brain vulnerability threshold framework in which the gut microbiota functions as one among several systemic modulators of neurological resilience, operating alongside genetics, aging, and environmental exposures. We do not posit the microbiota as a primary etiological agent of classical neurodegenerative diseases. Rather, microbiota-derived inflammatory and metabolic signals shape the systemic milieu that determines neuronal resilience or susceptibility. In genetically and epigenetically predisposed individuals, microbial alterations may lower the threshold for disease expression, functioning as modulators of risk rather than singular causal triggers. This reframing has direct implications for therapeutic strategy. If the microbiota modulates vulnerability rather than initiating disease, then microbiota-targeted interventions may delay onset, slow progression, and expand the therapeutic window, even in the absence of direct disease modification (Fig. 1).

### Distinguishing the brain vulnerability threshold framework from multi-hit models

The vulnerability threshold framework proposed here is related to but distinct from existing multi-hit models of neurodegeneration. Multi-hit models posit that neurological disease requires the accumulation of multiple independent pathogenic insults, each contributing additively or synergistically to disease onset. The vulnerability threshold framework makes a more specific and testable prediction: microbiota-derived signals do not constitute an independent pathogenic hit but instead modulate the quantitative threshold at which a given burden of neurological stress produces clinical disease expression. This distinction matters because it implies that microbiota-targeted interventions can shift disease onset trajectories and slow progression without eliminating any single causative factor, a hypothesis directly testable through longitudinal biomarker studies in prodromal populations stratified by microbiome composition and inflammatory tone. Furthermore, the framework generates specific predictions about which individuals are most likely to benefit from microbiota-targeted intervention, specifically those whose systemic inflammatory and metabolic state is closest to the clinical tipping point, providing a basis for precision stratification that multi-hit models do not explicitly offer. Whether this framework is ultimately validated, falsified, or refined will depend on prospective studies that measure microbiome composition, systemic inflammatory markers, and neurological endpoints simultaneously in large prodromal cohorts, an experimental design that the field has not yet executed at sufficient scale.

To facilitate reader orientation, key terms used throughout this review are defined in Table 1.

**Table 1:** Definitions of key terms

Term	Definition
Microbiota	The community of living microorganisms (bacteria, fungi, viruses, archaea) residing in a specific environment, such as the gastrointestinal tract.
Microbiome	The collective genetic material of the microbiota, including all microbial genes and their functional products.
Probiotic	Live microorganisms that, when administered in adequate amounts, confer a health benefit on the host.
Prebiotic	Non-digestible dietary substrates that selectively stimulate the growth or activity of beneficial gut microorganisms.
Postbiotic	Bioactive compounds produced by microorganisms during fermentation, or released upon cell lysis, that confer a health benefit when administered in adequate amounts.
Synbiotic	A combination of probiotics and prebiotics that act synergistically to improve host health by enhancing the survival and activity of beneficial microorganisms.
Short-Chain Fatty Acids (SCFAs)	Microbial fermentation products of dietary fiber, primarily acetate, propionate, and butyrate, that regulate immune tone, gut barrier integrity, and neuronal metabolism.
Fecal Microbiota Transplantation (FMT)	The transfer of fecal material from a healthy donor into the gastrointestinal tract of a recipient to restore microbial community composition.
Blood-Brain Barrier (BBB)	A selective semipermeable border of endothelial cells that regulates the passage of substances from the bloodstream into the central nervous system.
Altered Microbial Composition	Changes in the diversity, abundance, or functional capacity of the gut microbiota, previously referred to as dysbiosis; this review uses specific descriptors rather than this imprecise term.

A glossary of terms used throughout this review to ensure clarity and consistency for readers from diverse disciplinary backgrounds

## Mechanisms of gut-brain communication

The most reproducible mechanistic links between the microbiota and brain pathology involve regulation of basal inflammatory tone, receptor signaling thresholds, metabolic programming, and barrier integrity rather than disease-specific protein aggregation pathways. Communication between the gut microbiota and the brain occurs through multiple parallel systems that converge on these regulatory domains.

The vagus nerve provides a direct anatomical conduit between the intestinal environment and the central nervous system. Afferent fibers detect microbial metabolites, neurotransmitters, and inflammatory mediators [10]. Experimental studies demonstrate that vagotomy can abolish behavioral effects of specific probiotics and that vagal stimulation alters gut microbial composition [11], supporting bidirectional signaling. In Parkinson’s disease models, enteric α-synuclein accumulation has been proposed to propagate along vagal projections toward the brainstem [12]. A recent study further demonstrated that intestinal interoceptive dysfunction in aged animals drives cognitive decline by disrupting vagal afferent signaling to the brain [13], directly linking gut sensory input to age-associated memory impairment and providing mechanistic support for the vulnerability threshold framework in aging. However, such observations are best interpreted within a permissive framework in which peripheral inflammatory or proteostatic disturbances may influence central vulnerability, rather than establishing a singular route of disease initiation.

Microbial metabolites represent a second major communication axis. Short-chain fatty acids (SCFAs), generated by bacterial fermentation of dietary fiber, influence microglial maturation, neuroinflammatory tone, and neuronal energy metabolism [14]. Butyrate, in particular, functions as a histone deacetylase inhibitor, altering transcriptional programs related to synaptic plasticity and stress responses while also maintaining intestinal barrier integrity [15]. At the cellular level, butyrate signals through G protein-coupled receptors GPR109A and GPR43 expressed on microglia and intestinal epithelial cells, suppressing NF-kB-mediated inflammatory gene transcription and promoting BDNF expression, which supports synaptic maintenance and neuronal survival. Reduced abundance of SCFA-producing bacteria has been reported in Alzheimer’s and Parkinson’s disease cohorts [16], suggesting that diminished metabolite signaling may shift inflammatory thresholds rather than directly induce protein misfolding. It is important to note, however, that fecal SCFA measurements are imprecise and potentially misleading proxies for biologically active SCFA concentrations. Fecal levels reflect the net balance of microbial production, colonic absorption, and transit time and do not reliably represent portal or systemic concentrations that reach the brain or peripheral immune compartments. Interpretations of fecal SCFA data in the neurological disease literature should therefore be treated with appropriate caution, and future studies should prioritize measurements of circulating or tissue SCFA levels where mechanistic inference is intended.

Beyond SCFAs, microbiota-driven regulation of tryptophan metabolism alters the balance between serotonin production and activation of the kynurenine pathway [17]. In inflammatory states, increased kynurenine metabolism can generate quinolinic acid, an NMDA receptor agonist associated with excitotoxic stress [18], further linking peripheral immune activation to neuronal susceptibility.

The immune system constitutes a third major interface. Microbial products such as lipopolysaccharide (LPS) activate toll-like receptor 4 (TLR4), thereby inducing downstream activation of the NLRP3 inflammasome and secretion of IL-1beta and IL-18, which promote synaptic pruning, impair protein clearance by disrupting autophagosome-lysosome fusion, and contribute to sustained microglial activation states associated with neurodegeneration [19]. Circulating cytokines and immune mediators influence microglial activation states, modulating oxidative stress and synaptic remodeling [20]. Age-associated alterations in microbial composition contribute to chronic low-grade inflammation, or “inflammaging,” which reduces resilience across multiple neurodegenerative contexts [21]. Conversely, certain microbial species promote regulatory T-cell development and anti-inflammatory signaling [22], highlighting that microbiota-derived signals can either amplify or buffer inflammatory stress.

Barrier integrity represents a fourth critical node of regulation. The intestinal epithelium and the blood–brain barrier function as coordinated gatekeepers of systemic and neural homeostasis [23]. Altered microbial composition-associated reductions in tight junction proteins increase intestinal permeability, facilitating systemic immune activation that may, in turn, compromise blood-brain barrier function [24]. SCFAs normally reinforce barrier stability; their depletion therefore creates a dual vulnerability in peripheral and central compartments [25]. Germ-free models demonstrate that microbial absence alters blood-brain barrier permeability, reversible upon microbial reconstitution or metabolite supplementation [26], reinforcing the concept that microbial signaling calibrates rather than singularly drives neurobiological risk.

Collectively, these pathways converge on a shared principle: the gut microbiota influences the inflammatory, metabolic, and barrier thresholds that determine neuronal resilience. Rather than acting as primary initiators of disease, microbial signals appear to shape the systemic context in which neurodegenerative processes unfold.

## Microbiota and aging

Aging is associated with a progressive alteration in microbial composition, characterized by reduced microbial diversity, depletion of butyrate-producing Firmicutes, and expansion of pro-inflammatory Proteobacteria [27]. These compositional shifts are linked to chronic, low-grade systemic inflammation, termed “inflammaging”, driven in part by increased intestinal permeability and enhanced translocation of microbial products such as lipopolysaccharide [28]. Reduced short-chain fatty acid production may weaken the blood-brain barrier and alter microglial homeostasis, while elevated circulating cytokines may influence endothelial activation and neuroinflammatory signaling [29]. Together, these changes suggest that age-related alterations in the microbiota recalibrate inflammatory and metabolic thresholds that influence neuronal resilience. It is important to note, however, that the compositional changes observed in aged individuals are highly variable across cohorts and geographic regions and are substantially influenced by diet, medication use, and comorbidities common in elderly populations. Causal inference from cross-sectional data in aging populations is, therefore, limited, and the findings summarized here should be interpreted as consistent with a modulatory role rather than as establishing directionality.

Experimental manipulation of the aged microbiota supports this modulatory model. Transplantation of microbiota from young donors into aged mice has been reported to improve hippocampal neurogenesis and cognitive performance [30], whereas germ-free conditions alter lifespan trajectories and inflammatory profiles [31]. Rather than establishing microbiota as primary determinants of aging, these findings indicate that microbial communities can amplify or buffer age-associated inflammatory stress, thereby influencing the trajectory of brain aging. A recent study demonstrated that intestinal interoceptive dysfunction in aging drives cognitive decline through disrupted vagal afferent signaling to the brain [13], providing a specific mechanistic pathway through which age-related changes in gut sensory function may directly influence central cognitive circuits and supporting the relevance of gut-brain communication as a modulator of age-associated neurological vulnerability.

Centenarians exhibit distinct microbial signatures enriched in bacteria associated with anti-inflammatory metabolite and bile acid production [32], suggesting that preserved microbial function may contribute to resilience. Dietary patterns such as Mediterranean or fiber-rich diets are associated with cognitive preservation in older adults and are known to reshape microbial composition and metabolite output [33]. While regional dietary variation correlates with differences in microbiota structure and reported dementia prevalence, these associations are heavily confounded by socioeconomic factors, comorbidities, and medication use and require longitudinal validation before mechanistic conclusions can be drawn [34].

Aging, therefore, appears to represent a permissive biological context in which microbiota-derived inflammatory signals intersect with epigenetic drift and immunosenescence. Within this framework, microbial alterations do not initiate neurodegeneration but may stabilize maladaptive transcriptional and inflammatory states that lower the threshold for disease expression.

Sex further modulates this interaction. Hormonal signaling influences microbiota composition and immune tone across the lifespan, potentially contributing to sex-biased patterns observed in disorders such as Alzheimer’s and Parkinson’s disease. Within a vulnerability-threshold model, sex acts as a biological modifier of resilience rather than merely a demographic variable, implying that microbiota-targeted interventions should be stratified accordingly.

## The role of gut microbiota in brain tumor development and therapy

The vulnerability threshold framework proposed in this review applies not only to neurodegenerative disease but also to neuro-oncological conditions in which the systemic immune environment determines whether the brain microenvironment permits or suppresses tumor growth and therapeutic response. In brain tumors, microbiota-derived signals modulate the systemic immune and inflammatory threshold that shapes anti-tumor immunity within the tumor microenvironment, directly analogous to the inflammatory threshold modulation described in neurodegeneration. In other words, the microbiota does not cause brain tumors but shapes the immune context in which tumor progression and therapeutic response are determined.

Emerging evidence suggests that gut microbiota composition may influence brain tumor development and therapeutic responses through systemic immune modulation, although direct evidence in primary brain tumors remains more limited compared to peripheral cancers [35]. Glioblastoma patients exhibit distinct altered microbial composition, characterized by reduced microbial diversity and altered metabolite production, compared to healthy controls. Specific bacterial taxa correlate with tumor grade and patient survival, indicating a possible modulatory role of the microbiota in disease progression [36]. The mechanistic link appears to involve microbiota-regulated systemic immune tone, which shapes the tumor microenvironment: certain bacterial species promote the differentiation of regulatory T cells (Tregs) and myeloid-derived suppressor cells (MDSCs), which infiltrate brain tumors and dampen anti-tumor immunity [37]. Conversely, commensal bacteria that enhance CD8 + T cell priming and natural killer (NK) cell activity have been shown to improve survival in preclinical glioma models, suggesting that microbiota can influence the immune landscape to support anti-tumor responses [38].

While evidence from primary brain tumors is still sparse compared to peripheral cancers, studies suggest that glioblastoma-associated microbial composition may influence tumor progression. However, it remains unclear whether this altered microbial diversity is a causal driver or a metabolic consequence of corticosteroid use, seizure medications, and disrupted eating patterns, factors that are often present in brain tumor patients [39]. Future studies should aim to control these confounding variables to establish more direct causal links. Recent work has further explored how the gut microbiome may be exploited therapeutically in brain tumors, including by modulating immunotherapy responses and reducing treatment-related neurotoxicity, highlighting the translational potential of microbiota-targeted strategies in neuro-oncology [40].

Beyond immune modulation, gut bacteria also influence chemotherapy efficacy and neurotoxicity. Specific microbiota taxa metabolize chemotherapeutic agents, thereby altering drug bioavailability and systemic toxicity. Additionally, bacterial metabolites can modulate blood-brain barrier (BBB) permeability, thereby affecting drug penetration into brain tumors [41]. These findings suggest that microbiota play a key role in shaping the pharmacokinetics of chemotherapeutic agents, potentially influencing treatment outcomes.

Microbiota-driven inflammatory signals may also facilitate the development of brain metastases from peripheral tumors. By compromising the integrity of the blood-brain barrier and creating a more permissive microenvironment, altered microbial composition may facilitate the extravasation of circulating tumor cells [42]. This insight highlights the broader impact of the gut microbiota on brain tumor biology, not only through immune modulation but also by influencing the structural integrity of the blood-brain barrier.

These findings position the gut microbiota as a potential therapeutic target in neuro-oncology. Ongoing clinical trials are exploring the potential for microbiota modulation to enhance the efficacy of immunotherapy and reduce treatment-related neurotoxicity in patients with primary and metastatic brain tumors [43]. In this context, the tumor microenvironment provides a powerful analogy for how systemic inflammatory and immune pathways, driven by microbial composition, can modulate brain vulnerability, suggesting that microbiota may influence the progression of brain tumors through similar mechanisms of inflammatory remodeling and cellular signaling hijacking.

## Microbial diversity and viral neuroinflammation

Chronic viral infections represent a third context in which the vulnerability threshold framework applies. Just as microbiota-derived signals lower the neurological resilience threshold in aging and neurodegeneration, gut microbial alterations in the context of chronic viral infection amplify the neuroinflammatory burden that determines whether viral CNS exposure produces clinical cognitive impairment. The microbiota does not cause HIV-associated neurocognitive disorders or post-viral cognitive dysfunction but shapes the inflammatory environment in which viral neurological injury is expressed and sustained.

Chronic viral infections, particularly HIV, induce significant altered microbial composition, which contributes to persistent neuroinflammation through mechanisms distinct from direct viral invasion of the brain [44]. HIV infection causes substantial depletion of gut-associated CD4 + T cells, disrupting intestinal barrier integrity and promoting bacterial translocation, which persists despite effective antiretroviral therapy [45]. This translocation releases lipopolysaccharide and bacterial DNA fragments into the circulation, triggering systemic immune responses characterized by elevated inflammatory cytokine levels. These cytokines can cross the blood-brain barrier, activating microglia and astrocytes, which contribute to HIV-associated neurocognitive disorders, even in virally suppressed individuals [46].

Gut microbiota in HIV-infected individuals exhibit reduced diversity, decreased abundance of beneficial species such as Bacteroides and Lactobacillus, and increased abundance of pro-inflammatory Proteobacteria [47]. These microbial shifts correlate with immune activation markers and neurocognitive impairment, suggesting that the gut microbiota plays a key role in modulating the inflammatory landscape that underlies cognitive dysfunction in HIV. Additionally, tryptophan metabolism in HIV patients shifts toward the kynurenine pathway, a neurotoxic metabolic route promoted by indoleamine 2,3-dioxygenase upregulation due to chronic microbial translocation. This shift leads to the production of quinolinic acid, which can cause excitotoxicity and neuronal damage [48].

Beyond HIV, other neurotropic viruses, including SARS-CoV-2, also induce altered microbial composition, which may contribute to neurological sequelae. COVID-19 patients exhibit altered microbiota composition that persists months after the acute infection phase and correlates with the emergence of neuropsychiatric symptoms, suggesting that microbiota-mediated inflammation could be a key mechanism of post-viral cognitive dysfunction [49]. Post-acute sequelae of SARS-CoV-2 infection (long COVID) provide a large-scale natural experiment that underscores the importance of microbiota in neuroinflammation. Persistent reduced microbial diversity six months after infection independently predicts neuropsychiatric symptom burden, highlighting the potential of microbiota normalization as an intervention target for post-viral cognitive dysfunction [50].

Interventions targeting gut health in viral infections have shown promise in modulating inflammatory responses. Probiotic supplementation in HIV patients reduces markers of microbial translocation and systemic inflammation, while dietary fiber intake increases short-chain fatty acid production, thereby dampening inflammatory signaling and potentially preserving cognitive function [50]. These findings underscore the gut microbiota’s critical role in linking chronic viral infections to neuroinflammation. However, rather than acting as direct causes of neurological disorders, viral infections may function as transient amplifiers of pre-existing inflammatory pathways conditioned by microbiota composition. This supports the concept of a multi-hit model of neurodegeneration, in which viral infection acts as a trigger but not a singular cause.

## Microbial composition in Alzheimer’s disease

Alzheimer’s disease (AD), the most common cause of dementia, is characterized by progressive memory decline and accumulation of amyloid-β (Aβ) plaques and hyperphosphorylated tau tangles [51]. Comparative microbiome studies consistently report reduced microbial diversity in AD patients, decreased abundance of short-chain fatty acid (SCFA)-producing Firmicutes, and expansion of pro-inflammatory Proteobacteria [52]. Several taxa show reproducible associations across cohorts, including increased Escherichia/Shigella and reduced Eubacterium, Roseburia, and Faecalibacterium [53]. It is important to note that these findings are not universally replicated across independent cohorts and are subject to substantial confounding by diet, medication use (including proton pump inhibitors and metformin), geographic variation, and the possibility that altered microbial composition reflects rather than precedes disease-associated changes in dietary behavior and physical activity. While these findings establish correlation rather than causation, they support a reproducible pattern of microbial alteration linked to inflammatory tone.

Bacterial production of functional amyloids has further expanded mechanistic hypotheses. Enteric bacteria such as Escherichia coli produce curli fibers, amyloid-like proteins that facilitate biofilm formation [54]. These structures share cross-β sheet architecture with human Aβ and can promote aggregation in experimental systems [55]. Exposure to bacterial amyloids enhances protein aggregation phenotypes in animal models, and microbial components have been detected in association with amyloid deposits in human tissue [56]. Additionally, bacterial lipopolysaccharide (LPS) can interact with Aβ and influence aggregation kinetics in vitro [57]. Germ-free APP transgenic mice exhibit reduced amyloid burden compared with conventionally colonized controls, and colonization alters pathology [58]. Collectively, these observations suggest that microbial products can modulate amyloid dynamics under permissive conditions, though they do not establish microbial signals as initiating events in human AD.

Neuroinflammation represents a more reproducible interface. Elevated circulating LPS and inflammatory cytokines correlate with cognitive decline and amyloid burden in patients [59]. Chronic systemic inflammation influences microglial activation states, shifting cells from homeostatic to pro-inflammatory phenotypes associated with oxidative stress and impaired synaptic support [60]. Pro-inflammatory cytokines such as IL-1β and TNF-α can promote tau phosphorylation and synaptic dysfunction [61], linking peripheral immune tone to central proteostatic stress. In this framework, amyloid and tau accumulation may reflect downstream manifestations of sustained inflammatory pressure rather than isolated primary triggers. Longitudinal studies indicating that inflammatory and metabolic disturbances precede overt cognitive decline [62] are consistent with a model in which gut-derived immune signals lower the threshold for pathological protein accumulation in vulnerable individuals.

Memory impairment, the defining clinical feature of AD, may therefore reflect cumulative effects of altered metabolite signaling and inflammatory tone on hippocampal resilience. Reduced SCFA availability is associated with impaired neurogenesis and altered microglial function [63]. Dysregulated tryptophan metabolism shifts the balance toward kynurenine pathway metabolites that can induce excitotoxic stress [64]. Altered bile acid profiles further influence lipid metabolism and synaptic signaling pathways relevant to plasticity [65]. Interventional studies in animal models and early clinical cohorts suggest that microbiota modulation can influence cognitive performance [66]; however, these findings should be interpreted as evidence of modulatory potential rather than disease reversal.

## Microbial composition in Parkinson’s disease

Parkinson’s disease (PD) affects over 10 million people worldwide and primarily manifests as progressive motor dysfunction, including tremor, rigidity, and bradykinesia, due to the loss of dopaminergic neurons in the substantia nigra and the accumulation of α-synuclein-containing Lewy bodies [67]. The connection between the gut microbiota and PD has drawn increasing attention following observations that constipation often precedes motor symptoms by years and that Lewy pathology is detectable in enteric neurons long before brain involvement [68]. Comprehensive sequencing studies reveal that PD patients exhibit distinct gut microbiota profiles, characterized by reduced Prevotella species, important for mucin degradation and SCFA production, and increased Enterobacteriaceae, which are linked to intestinal inflammation [69]. The extent of Prevotella depletion correlates with motor symptom severity, suggesting that gut microbial alterations influence disease progression rather than merely correlating with it [70], though this interpretation requires longitudinal confirmation.

The “gut-first” hypothesis of PD pathogenesis posits that α-synuclein misfolding may initiate in enteric neurons and propagate to the brain via the vagus nerve. This model, supported by Braak staging, shows that Lewy pathology first appears in the gut and lower brainstem before spreading to the substantia nigra [71]. Experimental studies in animals have demonstrated that injecting preformed α-synuclein fibrils into the intestinal wall triggers a time-dependent spread of pathology via vagal projections to the brainstem and, eventually, to the substantia nigra, mimicking the progression observed in human PD [72]. Truncal vagotomy, performed decades before PD onset, is associated with reduced PD risk in epidemiological studies, though this protection is not observed when the gastric branch of the vagus is spared [73]. These findings suggest that the vagus may serve as a conduit for α-synuclein propagation, but the precise role of microbiota in this process and the extent to which it applies to sporadic human PD remain to be fully clarified.

A key metabolic consequence of PD-associated what has been termed dysbiosis, though this term carries limitations, is the deficiency of SCFAs, particularly butyrate. Reduced fecal butyrate concentrations in PD patients correlate with disease severity and progression [74], though as noted above, fecal SCFA levels are imprecise proxies for biologically relevant concentrations. Since butyrate is a primary energy source for colonocytes and plays a crucial role in maintaining intestinal barrier integrity, its deficiency contributes to increased intestinal permeability and subsequent systemic inflammation in patients with PD [75]. Studies in germ-free α-synuclein-overexpressing mice have shown that these animals exhibit reduced motor deficits and neuroinflammation compared with conventionally housed controls. Colonization with microbiota from PD patients, but not healthy controls, exacerbates motor symptoms and brain pathology. Treatment with SCFAs, on the other hand, reversed these deficits, demonstrating that microbial metabolites can modulate PD progression through neuroprotective mechanisms, including activation of GPR43 receptors that dampen microglial activation and enhance dopaminergic neuron energy metabolism [76]. PD is classically considered a movement disorder, but cognitive decline and dementia occur in up to 80% of patients after 20 years of disease progression [77]. Reduced microbial diversity contributes to cognitive impairment through neuroinflammation that affects not only the substantia nigra but also cortical and hippocampal regions crucial for memory and executive function [78]. In PD, depletion of butyrate-producing bacteria like Roseburia correlates with accelerated cognitive decline and earlier onset of dementia [79]. This neurodegeneration likely arises from both direct effects of microbial metabolites on synaptic plasticity and indirect effects driven by systemic inflammation that disrupts hippocampal neurogenesis and long-term potentiation, key processes for memory formation and cognitive flexibility [80].

## Gut composition in amyotrophic lateral sclerosis

Amyotrophic lateral sclerosis (ALS), the most common motor neuron disease, affects approximately 450,000 people globally, leading to the progressive degeneration of upper and lower motor neurons, paralysis, and death typically within 3–5 years of symptom onset [81]. While ALS was traditionally considered purely neurological, emerging evidence indicates significant alterations in gut microbiota composition that correlate with ALS disease progression, though the causal relationship remains unclear and findings are subject to substantial methodological variability across studies [82]. Metagenomic sequencing studies show reduced overall microbial diversity, and specific decreases in anti-inflammatory bacterial genera, including Butyrivibrio, Oscillibacter, and Anaerostipes [83]. The severity of altered microbial composition correlates with functional decline, as measured by the ALS Functional Rating Scale-Revised, suggesting that microbiota composition may serve as a prognostic marker; however, whether these changes precede or follow disease progression remains to be determined through prospective validation [84].

A prominent feature of ALS pathogenesis is metabolic dysfunction, including hypermetabolism despite muscle wasting, and alterations in gut microbiota contribute to this metabolic imbalance [85]. A study by Blacher and colleagues identified nicotinamide metabolism as a critical microbiota-regulated pathway influencing motor neuron survival [86]. In ALS patients, reduced bacterial capacity to synthesize nicotinamide from dietary precursors results in diminished NAD+ production, an essential molecule for neuronal energy metabolism and DNA repair [87]. Supplementation with nicotinamide or its precursors has been shown to extend survival in ALS mouse models, and fecal microbiota transplantation (FMT) from healthy donors to ALS mice improved motor function and extended lifespan by restoring the abundance of nicotinamide-producing bacteria [88]. Furthermore, gut bacteria regulate branched-chain amino acid metabolism, and altered microbiota in ALS patients can exacerbate metabolic dysfunction and potentially contribute to glutamate-mediated excitotoxicity [89].

Immune system dysregulation is another critical feature of ALS, with mounting evidence that the balance between pro-inflammatory and regulatory immune cells influences disease progression [90]. ALS patients show decreased numbers and impaired function of Tregs, which normally suppress excessive inflammation, and increased IL-17-producing T cells that promote neuroinflammation [91]. The gut microbiota plays an important role in this immune balance by producing metabolites, including SCFAs, which support Treg differentiation and function [92]. Studies transferring ALS patient-derived microbiota into mice have demonstrated that reduced microbial diversity impairs Treg function and accelerates motor neuron degeneration, while reconstitution with specific SCFA-producing bacteria enhances Treg function and extends survival [93]. These findings suggest that microbiota-targeted interventions, such as probiotic supplementation, prebiotic fiber, or FMT, may hold therapeutic potential by restoring immune homeostasis and slowing ALS progression.

Although motor symptoms dominate the clinical course of ALS, cognitive and behavioral changes occur in approximately 50% of patients and reflect frontotemporal cortical dysfunction, sharing pathological features with frontotemporal dementia [94]. The C9orf72 hexanucleotide repeat expansion, the most common genetic cause of familial ALS, is frequently associated with cognitive impairment and behavioral disinhibition [95]. Recent studies demonstrate that gut microbiota composition differs between ALS patients with and without cognitive impairment, with greater altered microbial composition observed in those with cognitive decline [96]. Mechanistically, gut-derived inflammatory signals may contribute to cognitive dysfunction by affecting vulnerable cortical populations, including von Economo neurons in the anterior cingulate cortex, which degenerate in both ALS with cognitive impairment and frontotemporal dementia [97]. Memory deficits in ALS are likely influenced by both direct effects of neuroinflammation on hippocampal synaptic plasticity and indirect effects mediated by disrupted sleep, pain, and psychological stress resulting from progressive motor disability [98].

## Convergent mechanisms across neurodegenerative diseases

### Microbiota-driven inflammation and epigenetic modifications in neurodegeneration

Chronic inflammation alone is unlikely to be sufficient to initiate neurodegeneration; however, sustained inflammatory signaling may induce stable epigenetic modifications, including altered DNA methylation at neuroinflammatory gene loci and changes in histone acetylation states mediated by SCFA-dependent histone deacetylase inhibition, that alter neuronal resilience, synaptic plasticity, and proteostatic balance, effectively converting a transient insult into a long-lasting pathogenic state. Emerging evidence reveals that AD, PD, and ALS share common microbiota-related pathological mechanisms, suggesting the potential for unified therapeutic strategies (Table 2). All three disorders exhibit reduced gut microbial diversity, depletion of SCFA-producing bacteria, and an increase in pro-inflammatory taxa, creating a recognizable pattern of microbial alteration across these neurodegenerative diseases [99]. This convergence reflects both the limited pathways through which the microbiota communicates with the brain and the shared neuronal vulnerability to inflammation, metabolic stress, and protein aggregation, irrespective of anatomical location or specific disease [100].

**Table 2:** Common Microbiota Alterations Across Neurodegenerative Diseases

Feature	Alzheimer’s Disease	Parkinson’s Disease	ALS	Shared Mechanism
Microbial Diversity	Decreased	Decreased	Decreased	Aging, diet, inflammation
SCFA-Producing Bacteria	Decreased (Eubacterium, Roseburia)	Decreased (Prevotella, Roseburia)	Decreased (Butyrivibrio, Oscillibacter)	Reduced fiber intake, inflammation
Fecal SCFA Levels	Reduced butyrate	Reduced butyrate, propionate	Reduced butyrate	Impaired bacterial fermentation
Pro-Inflammatory Bacteria	Increased (Escherichia, Shigella)	Increased (Enterobacteriaceae)	Increased (Proteobacteria)	Intestinal inflammation, barrier dysfunction
Plasma LPS Levels	Elevated	Elevated	Elevated	Intestinal permeability
Systemic Inflammation	IL-6, TNF-α, IL-1β elevated	IL-6, TNF-α elevated	IL-6, IL-17 elevated	Gut-derived immune activation
BBB Permeability	Increased	Increased	Increased	SCFA deficiency, inflammation
Protein Misfolding	Aβ, tau	α-synuclein	TDP-43, SOD1	Bacterial amyloid seeding, inflammation
Memory Impairment	Primary feature	Late feature	Subset of patients	Hippocampal neuroinflammation

Neurodegenerative disease-associated gut microbiota and systemic inflammatory alterations; *AD* Alzheimer’s disease, *PD* Parkinson’s disease, *ALS* amyotrophic lateral sclerosis, *SCFA* short-chain fatty acids, *BBB* blood–brain barrier, *LPS* lipopolysaccharide, *IL* interleukin, *TNF* tumor necrosis factor, *Aβ* amyloid-beta, *TDP-43* TAR DNA-binding protein 43, *SOD1* superoxide dismutase 1

A critical caveat is that shared microbial signatures across AD, PD, and ALS may reflect common consequences of neurodegeneration, dietary restriction, reduced mobility, and polypharmacy rather than upstream drivers of disease. Longitudinal microbiome profiling in presymptomatic populations, particularly in PD prodromal cohorts and individuals with genetic risk factors for AD and ALS, is necessary to establish directionality. The current literature is dominated by cross-sectional studies with small sample sizes, heterogeneous methodology, and inadequate control for confounding variables including diet, geographic region, medication use, and physical activity. Several associations reported in early studies have not replicated across independent cohorts, underscoring the need for methodological standardization and larger prospective studies before mechanistic or therapeutic conclusions can be drawn with confidence.

### Bacterial amyloids and protein aggregation

Bacterial amyloid production represents a mechanistic link between altered microbial composition and protein aggregation in neurodegenerative diseases. Initially discovered in the context of AD and α-synuclein aggregation in PD, emerging evidence suggests that bacterial curli fibers and other microbial amyloids may also influence TDP-43 and SOD1 aggregation in ALS [101]. The structural similarity between bacterial and human amyloid proteins enables cross-seeding, where bacterial amyloid templates the misfolding of human proteins that would otherwise remain soluble [102]. This process may help explain why germ-free or antibiotic-treated animals show reduced protein aggregation across multiple disease models despite the continued expression of aggregation-prone human proteins [103].

### Neuroinflammation and microglial activation

Neuroinflammation driven by gut-derived signals creates a permissive brain environment for neurodegeneration, regardless of the initial insult. Microglia, the brain’s primary inflammatory cells, exist along a spectrum of activation states, ranging from those that support neuronal health to those that produce neurotoxic mediators in an inflammatory phenotype [104]. Reduced microbial diversity shifts microglia toward pro-inflammatory activation through multiple pathways, including LPS-TLR4-NLRP3 inflammasome signaling, reduced SCFA-mediated suppression of GPR109A and GPR43, leading to NF-κB activity, and peripheral immune cell infiltration via a compromised blood-brain barrier [105]. This inflammatory milieu damages neurons through oxidative stress and excitotoxicity and impairs protein clearance mechanisms, including autophagy and proteasomal degradation, thereby allowing toxic protein aggregates to accumulate [106].

The chronic nature of inflammation stemming from persistent reduced microbial diversity distinguishes this process from acute inflammatory responses, which typically resolve once the triggering insult is removed. Instead, sustained inflammatory stress overwhelms compensatory mechanisms, accelerating neurodegeneration.

### Memory dysfunction and hippocampal vulnerability

Memory dysfunction, a hallmark feature of AD, occurs to varying degrees in PD and ALS, with common microbiota-dependent mechanisms contributing to cognitive decline. The hippocampus, essential for memory formation and spatial navigation, is particularly vulnerable to gut-derived inflammatory signals. Systemic administration of LPS impairs hippocampal long-term potentiation and spatial memory, even in young, healthy animals, highlighting the impact of microbiota-driven inflammation on cognitive function [107]. Chronic alterations in microbial composition reduce hippocampal neurogenesis, inhibiting the generation of new neurons in the dentate gyrus, which is critical for memory formation and cognitive flexibility. This occurs through mechanisms involving reduced SCFA-mediated histone acetylation and increased inflammation, which inhibit neural progenitor cell proliferation [108].

Additionally, the gut microbiota regulates the synthesis and metabolism of neurotransmitters, including serotonin, dopamine, GABA, and acetylcholine, thereby modulating synaptic plasticity and memory consolidation in the hippocampus [109]. Studies showing that probiotic supplementation improves memory performance in aged mice and patients with mild cognitive impairment suggest the therapeutic potential of modulating the microbiota to preserve cognitive function.

## Therapeutic interventions targeting the microbiota-gut-brain axis

The recognition that reduced microbial diversity contributes to neurodegeneration has catalyzed the development of microbiota-directed therapeutic strategies ranging from dietary interventions to fecal microbiota transplantation (Table 3). Probiotic supplementation with specific bacterial strains demonstrating neuroprotective properties in preclinical models represents the most accessible intervention currently available. Multiple randomized controlled trials in patients with AD have tested combinations of Lactobacillus and Bifidobacterium species, with several showing improvements in cognitive performance, as measured by Mini-Mental State Examination scores [110]. A 12-week trial of probiotic milk containing Lactobacillus acidophilus, L. casei, Bifidobacterium bifidum, and L. fermentum demonstrated significant improvements in metabolic parameters and modest cognitive benefits in AD patients compared to placebo. Similarly, PD patients receiving multi-strain probiotics for 12 weeks showed improvements in constipation, quality-of-life scores, and inflammatory markers, though motor symptoms remained largely unchanged [111]. The mechanisms underlying these benefits likely involve the restoration of intestinal barrier integrity, reduced systemic inflammation, and increased production of beneficial metabolites, including SCFAs and neurotransmitter precursors.

**Table 3:** Microbiota-Targeted Therapeutic Strategies in Neurological Disease

Intervention	Mechanism	Clinical Evidence	Advantages	Limitations
Probiotics	Restore beneficial bacteria, produce SCFAs, reduce inflammation	AD: modest cognitive improvement; PD: improved constipation and quality of life; ALS: ongoing trials	Widely available, safe, low cost	Strain-specific effects, variable colonization, modest efficacy
Prebiotics (dietary fiber)	Enhance SCFA production, promote beneficial bacteria	Mediterranean diet improves cognition in elderly; high-fiber diets associated with reduced AD risk	Diet-based, sustainable, additional health benefits	Requires dietary compliance, slow onset, individual variation
Synbiotics	Combined prebiotic + probiotic effects	Limited data in neurodegeneration; promising in metabolic disorders	Potential synergistic benefits	Increased complexity, higher cost
Fecal Microbiota Transplant	Complete microbiome replacement	PD: small trials show motor improvement; AD/ALS: case reports only	Rapid microbiome normalization	Safety concerns, standardization issues, regulatory barriers
Postbiotics (metabolites)	Direct delivery of beneficial metabolites (e.g., butyrate, nicotinamide)	Butyrate improves memory in AD mice; nicotinamide extends survival in ALS mice; clinical trials ongoing	Defined composition, direct mechanism	Dosing unclear, delivery challenges, limited human data
Dietary interventions	Modify microbiota composition through food choices	Mediterranean and MIND diets associated with cognitive benefits in longitudinal studies	Holistic approach, multiple health benefits, sustainable	Adherence challenges, slow effects, confounding by other dietary factors

Summary of current and emerging microbiota-directed interventions with clinical evidence, advantages, and limitations; *AD* Alzheimer’s disease, *PD* Parkinson’s disease, *ALS* amyotrophic lateral sclerosis, *SCFA* short-chain fatty acids, *QOL* quality of life, *MIND* Mediterranean-DASH Intervention for Neurodegenerative Delay

Prebiotic interventions using dietary fiber and resistant starches that selectively promote the growth of beneficial bacteria offer a complementary approach to probiotic supplementation. Inulin, fructo-oligosaccharides, and galacto-oligosaccharides increase the abundance of Bifidobacterium and Lactobacillus species while enhancing SCFA production [112]. The Mediterranean diet, rich in fruits, vegetables, whole grains, and olive oil, is associated with reduced AD risk and slower cognitive decline in longitudinal studies, with effects partly mediated by favorable modulation of gut microbiota composition [113]. The Dietary Approaches to Stop Hypertension (DASH) diet and Mediterranean-DASH Intervention for Neurodegenerative Delay (MIND) diet, specifically designed to reduce dementia risk, promote fiber intake, and plant-based foods that support beneficial gut bacteria [114].

Fecal microbiota transplantation, involving the transfer of entire microbial communities from healthy donors to patients, represents the most dramatic microbiota intervention, with growing evidence supporting its use in neurological disorders. Small pilot studies in PD patients undergoing colonoscopic FMT from healthy donors reported improvements in motor symptoms, constipation, and quality-of-life measures, sustained for up to 1 year [115]. The mechanisms likely involve rapid restoration of SCFA-producing bacteria, normalization of inflammatory markers, and correction of metabolic abnormalities that developed over years of progressive reduced microbial diversity. However, significant safety concerns remain following a death in an immunocompromised patient receiving FMT contaminated with pathogenic bacteria, highlighting the need for rigorous donor screening and preparation protocols [116]. Additionally, the optimal route of administration [colonoscopic, nasogastric, oral capsules], frequency of treatment, and patient selection criteria require systematic investigation in properly designed clinical trials [117].

Postbiotic approaches that deliver specific beneficial metabolites rather than live bacteria offer potential advantages, including a defined composition, easier regulation, and the ability to achieve therapeutic concentrations that may not be attainable through bacterial production alone. Sodium butyrate supplementation in AD mouse models improves memory performance, reduces amyloid pathology, and enhances hippocampal histone acetylation [118]. Human trials testing butyrate supplementation for cognitive enhancement are underway. Similarly, supplementation with nicotinamide or its precursors, whose production is impaired in ALS-associated microbiota composition, extends survival in ALS mice and is being tested in human trials [119]. Tryptophan metabolism can be modulated by supplementing with specific amino acids or by inhibiting the kynurenine pathway to redirect tryptophan toward serotonin synthesis rather than neurotoxic quinolinic acid production [120].

## Future directions and conclusions

Future progress will depend on clearly distinguishing microbial modulation of inflammatory and epigenetic states from direct etiological claims. Moving the field beyond associative correlations requires mechanistic stratification of disease vulnerability rather than binary causality models. A central unresolved question is whether microbiota-driven inflammatory signals can induce durable epigenetic reprogramming in neurons or glia, including altered DNA methylation dynamics and chromatin remodeling mediated by factors such as MeCP2. If such changes occur, they may provide a mechanistic basis for persistent susceptibility to disease long after the initiating peripheral trigger has resolved.

Cross-study interpretation remains limited by substantial methodological heterogeneity. 16 S rRNA sequencing captures taxonomic composition but not functional capacity; geographic, dietary, and demographic variables differ across cohorts; and the absence of standardized biobanking and processing protocols in neurological studies limits reproducibility and meta-analytic reliability. Several microbiome associations reported in early studies have not replicated in independent cohorts, and the field requires prospective longitudinal studies with standardized methodology and pre-specified endpoints before definitive mechanistic or therapeutic conclusions can be drawn.

Confounding represents a particularly serious interpretive challenge that has been insufficiently addressed in the existing literature. Diet is the dominant determinant of microbiome composition and is itself strongly and independently associated with neurological risk, making it extremely difficult to attribute observed microbiome-disease associations to microbial mechanisms rather than dietary effects. Medication use, including proton pump inhibitors, metformin, antipsychotics, and antibiotics, substantially alters microbiome composition and is highly prevalent in the populations studied. Geographic and ethnic variation in microbiome structure is large relative to disease-associated differences. Physical activity and mobility, which differ substantially between patients and controls in ALS and PD studies, independently alter gut transit and microbial composition. Future studies must prospectively collect and statistically control for these variables rather than treating them as nuisance factors in post-hoc sensitivity analyses.

Therapeutic translation must therefore proceed cautiously. Determining optimal strategies, whether probiotics, prebiotics, dietary modulation, or fecal microbiota transplantation, requires rigorously designed comparative trials with standardized endpoints and mechanistic readouts. Precision approaches that account for inter-individual microbiome variability will likely be necessary, as uniform interventions are unlikely to yield consistent outcomes across heterogeneous populations.

Importantly, the gut microbiota represents a modifiable interface rather than a primary driver of classical neurodegenerative diseases. Unlike genetic risk or chronological aging, microbial composition can be altered through diet, pharmacological agents, and microbial interventions. This accessibility justifies investigation of microbiota-targeted strategies in prodromal or high-risk individuals; however, such interventions should be viewed as modulatory components within broader preventive frameworks rather than disease-defining therapies.

Ultimately, progress in this field requires reframing microbiota–brain interactions within an integrative model that accounts for aging, host genetics, systemic inflammation, and environmental exposures. Recognizing the microbiota as environmental modulators rather than singular etiological agents will prevent conceptual overreach while preserving the biological plausibility of their contribution to neurodegenerative vulnerability.

## Data Availability

Not applicable. This manuscript is a review article and does not report original data.
